# Ideal number of biopsy tumor fragments for predicting HER2 status in gastric carcinoma resection specimens

**DOI:** 10.18632/oncotarget.5368

**Published:** 2015-10-09

**Authors:** Sangjeong Ahn, Soomin Ahn, Michael Van Vrancken, Minju Lee, Sang Yun Ha, Hyuk Lee, Byung-Hoon Min, Jun Haeng Lee, Jae J. Kim, Sunkyu Choi, Sin-Ho Jung, Min Gew Choi, Jun-Ho Lee, Tae Sung Sohn, Jae Moon Bae, Sung Kim, Kyoung-Mee Kim

**Affiliations:** ^1^ Department of Pathology & Translational Genomics, Samsung Medical Center, Sungkyunkwan University School of Medicine, Seoul, Korea; ^2^ Center for Companion Diagnostics, Innovative Cancer Medicine Institute, Samsung Medical Center, Seoul, Korea; ^3^ Department of Medicine, Samsung Medical Center, Sungkyunkwan University School of Medicine, Seoul, Korea; ^4^ Biostatistics and Clinical Epidemiology Center, Samsung Medical Center, Sungkyunkwan University School of Medicine, Seoul, Korea; ^5^ Department of Surgery, Samsung Medical Center, Sungkyunkwan University School of Medicine, Seoul, Korea; ^6^ Present address: Department of Pathology, Pusan National University Hospital and Pusan National University School of Medicine and BioMedical Research Institute, Pusan National University Hospital, Busan, Korea

**Keywords:** stomach, biopsy, operation, HER2, immunohistochemistry

## Abstract

Intratumoral heterogeneity of HER2 expression is common in gastric cancers and pose a challenge for identifying patients who would benefit from anti-HER2 therapy. The aim of this study is to compare HER2 expression in biopsy and resection specimens of gastric carcinoma by immunohistochemistry (IHC) and to find the ideal number of biopsy tumor fragments that can accurately predict HER2 overexpression in the corresponding surgically resected specimen. The HER2 IHC results of 702 paired biopsy and resection specimens of gastric cancer were compared.

The mean number of biopsy fragments among all cases was 4.3 (range 1–11). HER2 was positive in 130 (18.5%) endoscopic biopsies and in 102 (14.5%) gastrectomy specimens. Intratumoral heterogeneity of HER2 was found in 80 (61.5%) biopsies and 70 (68.6%) resection specimens. Out of the 70 surgical specimens with intratumoral heterogeneity, 24 (34.3%) of the corresponding biopsies were categorized as negative (positive conversion). In the 86 (12.3%) discrepant cases, negative conversion was observed in 57 (66.3%) cases and positive conversion in 29 (33.7%). The fragment numbers were significantly correlated with the discrepancy of results and positive predictability (*P* = 0.0315 and *P* = 0.0052). ROC curve analysis and positive predictability showed that 4 fragments should be obtained to minimize the differences in HER2 scores between biopsy and resection specimen.

In gastric carcinomas with discrepant HER2 results between biopsy and surgical resection specimens, intratumoral heterogeneity is common with most of them showing positive conversion. To predict HER2 status precisely, at least 4 biopsy fragments containing tumor cells are required.

## INTRODUCTION

Since its first expression characteristics were described in gastric cancer in 1986, human epidermal growth receptor 2 (HER2) has become an established predictive biomarker in this disease [1, 2]. Subsequent studies have shown that HER2 expression is more commonly associated with intestinal-type morphology, higher proliferation rates, proximal gastric location, and advanced tumor stage [3, 4]. Recently, HER2 targeted therapy has been shown to benefit patients with HER2-positive gastric cancers [5]. Additionally, this positive response is correlated with higher levels of HER2 expression [6, 7]. Therefore, an accurate assessment of HER2 in gastric cancer is of paramount importance for characterization and appropriate use of anti-HER2 therapy.

However, determining HER2 expression status in the practical setting presents a few challenges. One challenge is due to tissue sampling in biopsy specimens compared with the resection specimens. To ameliorate diagnostic accuracy and reduce discordance between biopsy and resection specimens, sufficient biopsy material is required. Several studies have compared HER2 results in paired biopsy and resection specimens and showed an overall concordance rate varying between 87 to 96% [8–15]. Several additional studies have proposed an optimal number of diagnostic biopsy fragments [8, 15] as well as a tumor fragment ratio to help decrease discrepant results [14]. However, these studies were limited as they were unable to find large numbers of discrepant cases of matched biopsy and resection specimens warranting further study with a much larger sample size. Ruschoff *et al* [16]. have recommended six to eight biopsy fragments in gastric cancer for HER2 testing while our group [17] has proposed four to six as acceptable. However, those recommendations are based on personal experiences and not a rigorous scientific quantitative assessment. Therefore, finding the ideal fragment number of biopsy specimens would be beneficial to accurately predict HER2 status, particularly when only endoscopic biopsy samples are available due to inoperability, which is frequently encountered in HER2-positive gastric cancers.

Another challenge associated with interpreting HER2 status in gastric cancer is the protein's affinity for being heterogeneously expressed. High incidence of HER2 heterogeneity is found in up to 79.3% of HER2-positive gastric cancers within the same tumor (‘intratumoral heterogeneity’) [9] and in up to 11% between primary gastric cancers and metastatic tumors (‘intertumoral heterogeneity’) [18]. Intratumoral heterogeneity is considered the main reason for discordant results between biopsy and resection specimens [8–13, 15]. Many oncologists and pathologists recognize intratumoral heterogeneity of HER2 and sufficient sampling is very important part of HER2 testing [19–22]. To determine the optimal number of endoscopic biopsies to predict HER2 status in gastric cancer, HER2 IHC results between the biopsy and gastrectomy specimens were compared.

## MATERIALS AND METHODS

### Patients

Only patients with primary gastric carcinoma who underwent both preoperative endoscopic biopsy and gastric cancer resection at Samsung Medical Center in Seoul, Korea from January 2013 to December 2014 were selected for this study. Patients who underwent preoperative chemotherapy and/or radiotherapy, or patients who were diagnosed with multiple gastric cancers were excluded. The clinicopathologic characteristics of 702 patients are described in Table 1. The median patient age was 61 years (range 22–88 years), and the male/female ratio was 1.9:1 (456 males and 246 females).

**Table 1:** Patients characteristics

*Characteristics*		*Number of cases (%)*
**Age**	Median (range)	61 (22–88)
**Gender**	Male Female	456 (65.0) 246 (35.0)
**Location**	Lower Middle Upper Whole	342 (48.7) 208 (29.6) 126 (17.9) 26 (3.7)
**Histologic type by Lauren**	Intestinal Mixed Diffuse Other	233 (33.1) 111 (15.8) 347 (49.4) 11 (1.6)
**pTstage**	1 2 3 4	227 (32.3) 104 (14.8) 177 (25.2) 194 (27.6)
**pNstage**	0 1 2 3	304 (43.3) 122 (17.4) 108 (15.4) 168 (23.9)
**AJCC stage**	I II III IV	259 (36.9) 176 (25.0) 263 (37.5) 4 (0.6)

A tumor fragment was defined as a piece of tissue containing 10 or more viable tumor cells in an endoscopic biopsy specimen as previously described [23].

### Immunohistochemistry

**Figure 1:**
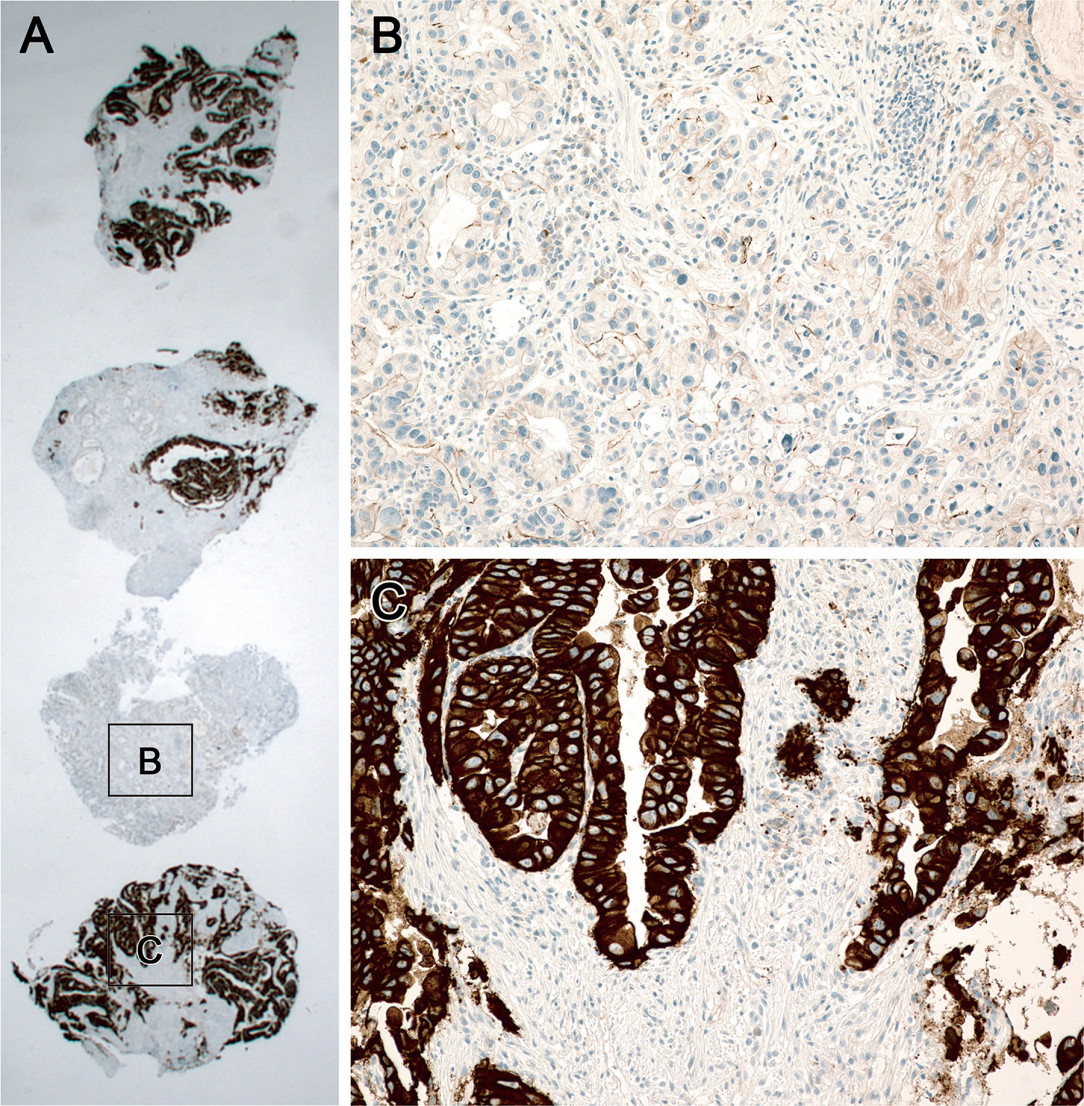
Heterogeneity of HER2 IHC staining in a biopsy specimen **A.** Four endoscopic biopsy fragments with tumor cells showing heterogeneous expression. One fragment (star) shows no staining in tumor cells **B.** while other three fragments stained strongly **C.**

**Figure 2:**
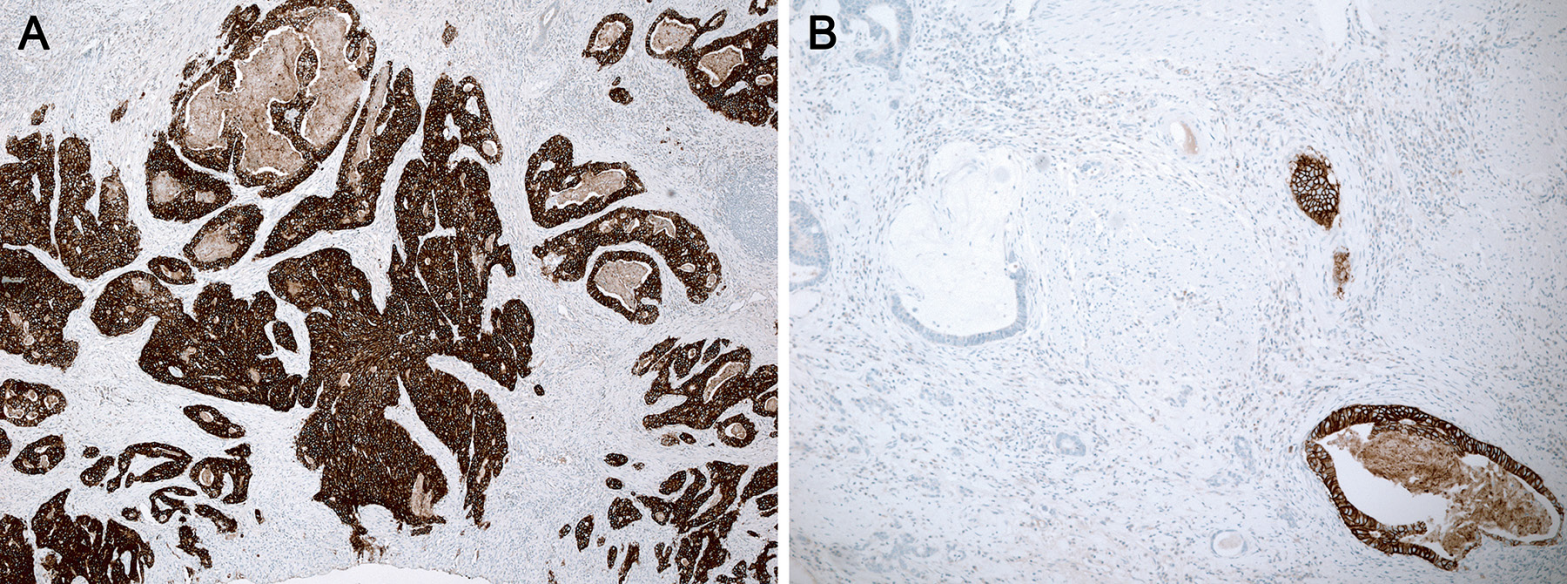
Heterogeneity of HER2 IHC staining in a resection specimen **A.** Homogeneous and **B.** heterogeneous membranous staining patterns were identified.

IHC for HER2 (PATHWAY HER-2/neu (4B5) rabbit monoclonal antibody, Ventana Medical Systems, Inc., Tucson, AZ) was performed in all cases with a BenchMark XT automated stainer (Ventana Medical Systems, Inc., Tucson, AZ). In operation specimens, we reviewed all hematoxylin and eosin stained tumor sections and selected a representative tumor block for IHC analysis. A gastrointestinal pathologist (KMK) evaluated the staining data with no previous knowledge of clinical or pathological parameters. HER2 IHC was scored according to the recently developed assessment guidelines for HER2-associated gastric cancers [17]. In biopsy specimens, intratumoral heterogeneity was defined as variable HER2 membranous staining among all submitted viable tumor fragments (Figure 1). In the resection specimens, it was defined as membranous staining in 10 ~ 90% of tumor cells (Figure 2). HER2 overexpression was defined as an IHC staining intensity of 2+ (equivocal) or 3+ [16].

### Statistical methods

A two-sample *T*-test was used to compare the number of total fragments between concordant (between biopsy and resection results) and discordant groups as well as between the biopsy positive and negative groups. Linear regression method was used to associate the difference in HER2 score between biopsy and resection specimen with the number of total fragments. A ROC curve was constructed to find a cutoff value for the number of total fragments. These analyses were conducted using SAS version 9.4 (SAS Institute, Cary, NC) and R 3.1.1 (Vienna, Austria; http://www.R-project.org) by biostatisticians (CS and JSH).

## RESULTS

### Comparison of HER2 results in paired biopsy and resection specimens

The IHC results of 702 paired biopsy and gastrectomy specimens were analyzed (Table 2). In biopsy specimens, 572 (81.5%) cases were negative for HER2 (0 or 1+), and 130 (18.5%) were positive (2+ or 3+). In surgical specimens, 600 (85.5%) were negative, and 102 (14.5%) were positive. The total number of positive cases in either biopsy or resection specimens was 159 (22.6%), and 3+ only cases was 63 (9.0%). Despite a relatively high concordance (*n* = 616, 87.7%) between biopsy and resection specimens, 86 (12.3%) paired specimens showed discrepant results. Of these discrepant cases, negative conversion (positive in biopsy and negative in resection) was found in 57 (66.3%) cases, and positive conversion was seen in 29 (33.7%) cases. After exclusion of equivocal 2+ cases, [24] the concordance rate increased to 95.9% (581 of 606 cases).

**Table 2:** HER2 IHC results in paired endoscopic biopsy and resection specimens

*Resection specimens*		*Biopsy specimens*					
		*Negative*		*Positive*		*Total*	*Concordance rate (%)*
		*0*	*1+*	*2+*	*3+*		
***Negative***	***0***	297	105	20	6	428	90.5
	***1+***	78	63	20	11	172	
***Positive***	***2+***	11	10	12	13	46	71.6
	***3+***	3	5	10	38	56	
	***Total***	389	183	62	68	702	87.7

### Intratumoral heterogeneity in paired biopsy and resection specimens

**Figure 3:**
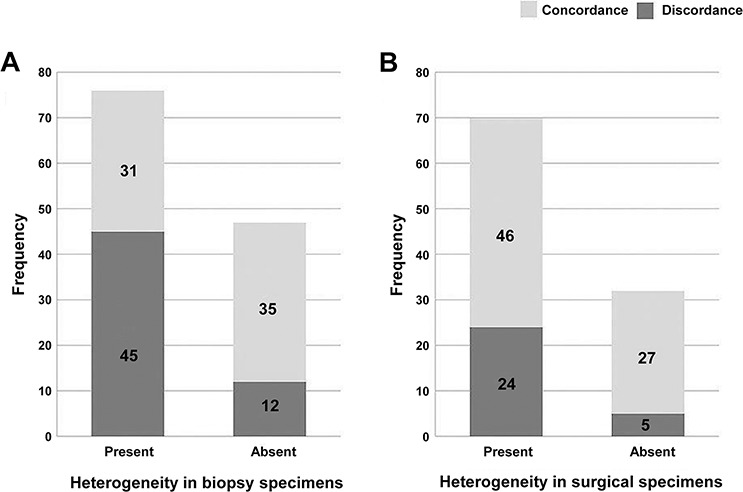
Heterogeneity in paired biopsy and resection specimens Cases with heterogeneity in either biopsy or operation specimens showed higher discrepant results compared to cases without heterogeneity (*P* = 0.0003 in biopsy, *P* = 0.0214 in operation).

Intratumoral heterogeneity was evaluated in 130 HER2-positive biopsy specimens and 80 cases showed intratumoral heterogeneity (61.5%). Out of 102 HER2-positive operation specimens, 70 cases (68.6%) showed heterogeneity, in which 24 (34.3%) showed discrepancy (positive conversion). Heterogeneity found in biopsy specimens was significantly correlated with its surgical operation specimen (*P* = 0.0116). Actually, for six cases with HER2 3+ in biopsies but negative on operation specimen, number of biopsy fragments were 2 (*n* = 1), 3 (*n* = 2), 4 (*n* = 2) and 5 (*n* = 1) and all five cases with > 2 biopsy fragments showed intratumoral heterogeneity within biopsy specimens. In those 6 cases, we further performed IHC using all tumor blocks from operation specimen and found 2+ in four cases and 3+ in two cases with positive tumor cells ranging from 1% to 10% of total tumor volume. Moreover, cases with heterogeneity in either biopsy or operation specimens showed higher discrepant results compared to cases without heterogeneity (*P* = 0.0003 in biopsy, *P* = 0.0214 in operation) (Figure 3). Our findings show that the heterogeneity seen in biopsy specimens likely predicts heterogeneity in the operation specimens. Additionally, intratumoral heterogeneity is the likely culprit that underlies the discrepant HER2 results between biopsy and operation specimens.

### Difference in HER2 scores in relation to the number of biopsy fragments

**Figure 4:**
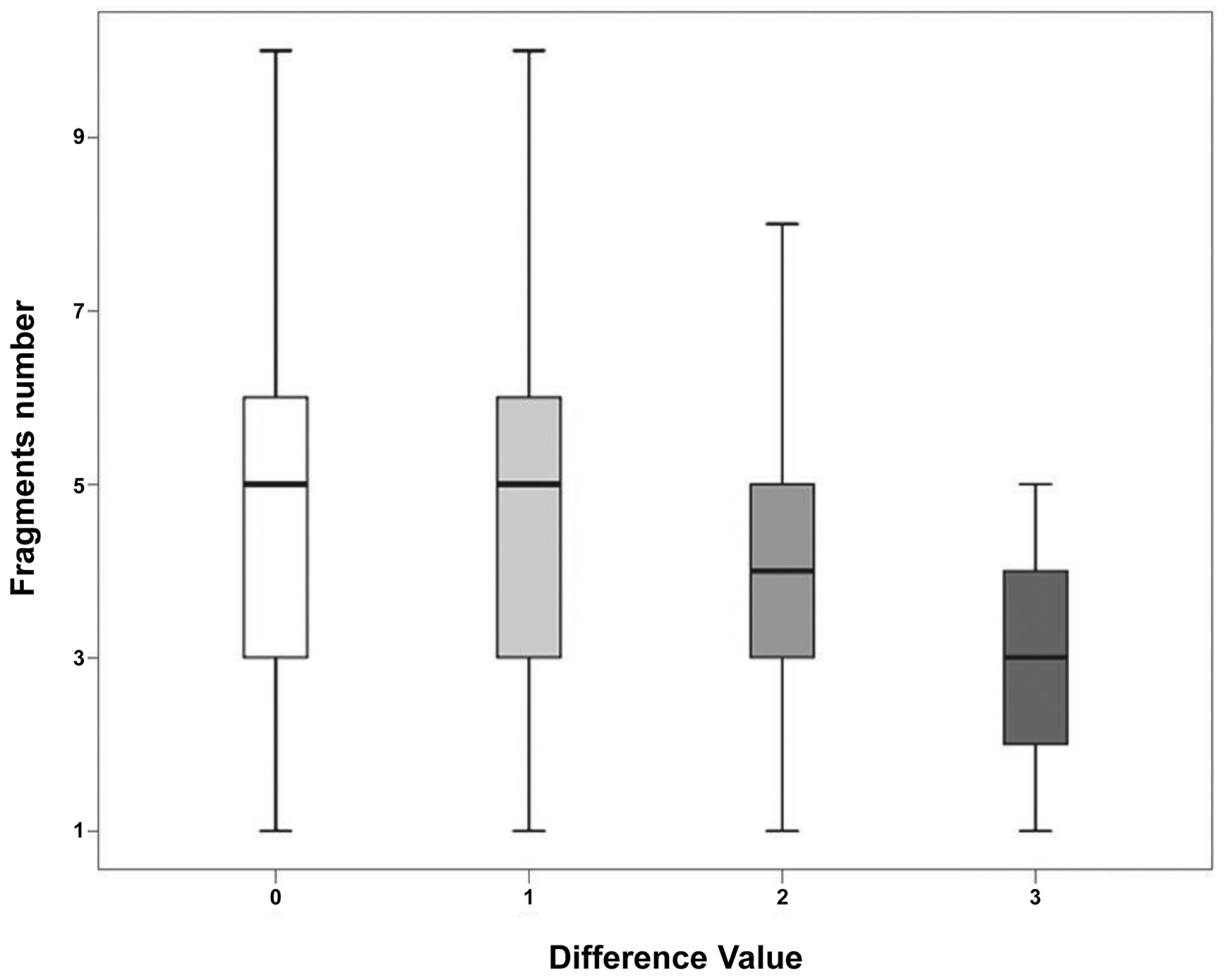
Difference in HER2 scores in relation to biopsy fragment numbers Fragment numbers were correlated with the difference (*P* = 0.0096) of HER2 expression score between the biopsy and resection specimen. The more biopsy fragments that are present, the less likely there is to be a difference (difference value of 0).

**Figure 5:**
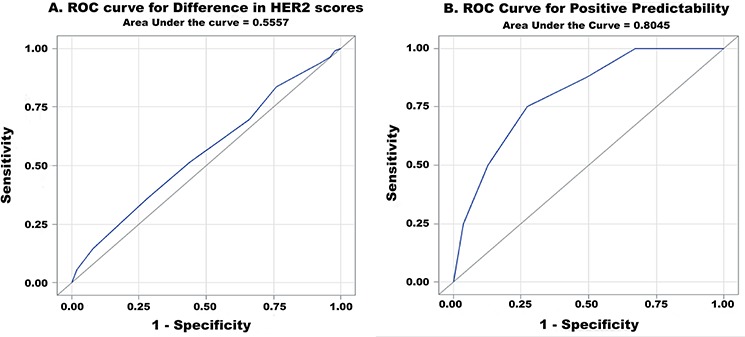
ROC curve analysis **A.** for difference in HER2 scores and **B.** for positive predictability (AUC = 0.5557 and 0.8045, respectively).

To assess the minimal requirement of biopsy fragments needed to accurately predict HER2 status, the difference in HER2 scores (HER2 score in resection specimen - HER2 score in the biopsy specimen) within the HER2-positive group was calculated (*n* = 159). This value was termed the “difference value”. For all paired specimens with a difference value of 0 (no difference between biopsy and resection HER2 status), the mean fragment count was 4.9 (*n* = 50). For the paired specimens with a difference value of 1, 2, and 3, the mean fragment count was 4.9 (*n* = 53), 4.3 (*n* = 47), and 2.9 (*n* = 9), respectively. Additionally, the number of biopsy fragments was significantly correlated with the difference value (*P* = 0.0096) (Figure 4). ROC curve analysis showed an area under curve (AUC) of 0.5557 with an ideal cut-off value of 3 (Figure 5A). Hence, 4 fragments should be recommended to minimize the difference in HER2 scores.

### Positive predictability in relation to the number of biopsy fragments

**Figure 6:**
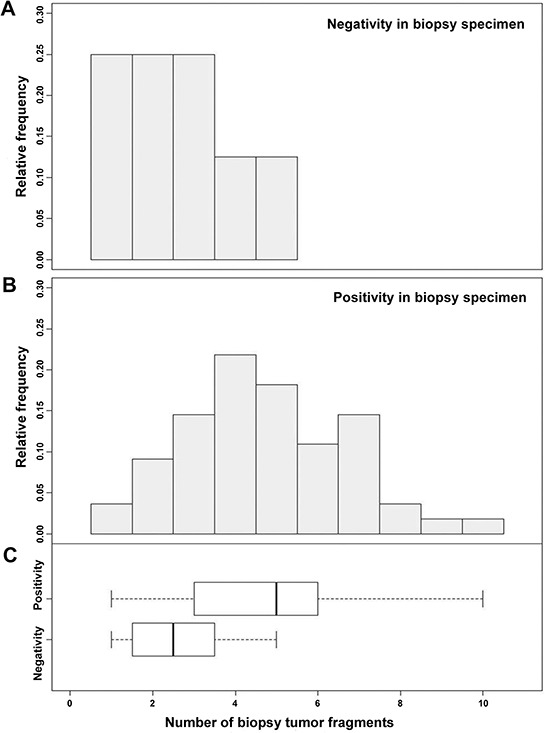
Positive predictability of HER2 status in biopsy specimens was significantly correlated with the number of fragments (*P* = 0.0052) For paired negative biopsy and positive resection specimens (positive conversion), the mean number of tissue fragments was 2.6 (range 1–5), while those positive in both biopsy and resection specimens had a mean biopsy fragment number of 4.7 (range 1–10).

‘Positive predictability’ was defined as the positive predictive value of HER2 in biopsy specimens. To assess the positive predictability in relation to the biopsy fragment numbers, fragment numbers within only the HER2 3+ positive group a (*n* = 63) in either biopsy or resection specimen were evaluated. HER2 equivocal (2+) cases were excluded to improve the precision of the calculation. Out of 63 cases, 8 cases showed positive conversion (negative in biopsy and positive in gastrectomy). The number of fragments in all positive conversion cases ranged from 1 to 5 (Figure 6) with a mean of 2.6. For the remaining HER2 3+ cases, the biopsy fragment range was 1 to 10 with a mean of 4.7. The positive predictability in biopsy specimens was significantly correlated with the number of biopsy fragments (*P* = 0.0052). According to ROC analysis, the ideal cut-off value was 3 with an AUC of 0.8045 (Figure 5B). Therefore, at least 4 fragments containing tumor should be recommended to maximize positive predictability in the biopsy specimens.

## DISCUSSION

Accurate prediction of HER2 status in endoscopic biopsies of gastric cancer is essential for accurate tumor characterization and also carries important therapeutic implications. However, the heterogeneity of HER2 overexpression in gastric cancer contributes to false-negative results in cases with limited biopsy material suggesting the necessity of extensive tissue sampling [11]. To our knowledge, this is the first study exploring the optimal number of endoscopic biopsy fragments to obtain in gastric cancer to assess HER2 status through IHC. This study has shown that the number of biopsy fragments is significantly correlated with HER2 status discrepancy between biopsy and operation specimens and also predicts positive predictability. Moreover, at least 4 biopsy fragments are necessary to minimize false-negative results and to reduce discrepancy between the biopsy and operation specimens.

Intratumoral heterogeneity is the major underlying cause of discrepant HER2 results between the biopsy and resection specimens [8–13, 15]. This study has shown that more endoscopic biopsy fragments helps predict intratumoral heterogeneity present in the subsequent resection specimens. To find the “optimal” number of biopsy fragments to predict HER2 status in gastric operation specimens, the actual difference value of HER2 scores identified between the paired biopsy and operation specimens was calculated among a large number of gastric cancer cases. The difference value, ranging from 0 to 3, was significantly correlated with fragment number, and further analysis showed that at least 4 fragments are required to maximize correlation between HER2 status of the biopsy and resection specimen. This study also suggests that the numbers of biopsy fragments containing tumor should be described in the HER2 IHC pathology report. In cases with negative HER2 IHC results with limited numbers of biopsy specimens (i.e., less than 3), re-testing HER2 IHC with more tissue sampling should be considered. In one prospective study of unresectable or metastatic gastric cancer which initially tested HER2 negative (Gasther-1 study), repeat endoscopic biopsy detected HER2 overexpression in which the initial biopsy missed [25].

In this study, the positive predictability of the biopsy specimen was also calculated. True positive cases were determined as HER2 3+ either in the biopsy or resection specimens. Out of 63 HER2 3+ cases, 12.7% of cases were negative in the biopsy specimen and positive in the resection specimen. Interestingly, all cases with positive conversion showed intratumoral heterogeneity in the surgical specimens confirming that heterogeneity is the major reason for the discordant results. In cases with positive conversion, the number of biopsy fragments ranged from 1 to 5. Endoscopic biopsies with six or more fragments showed 100% HER2 status correlation with the surgically resected specimen. In our statistical analyses, a minimum of 4 biopsy fragments was needed to accurately predict HER2 status in the resected specimens.

In summary, due to significant intratumoral heterogeneity, the larger the number of endoscopic biopsy fragments available for HER2 IHC analysis in gastric cancer, the higher the correlation with HER2 status will be in the resection specimen. The discordance increased with smaller numbers of biopsy fragments. This warrants some caution in relying on HER2 IHC findings of endoscopic biopsy specimens alone to determine treatment regimens.

Finally, we suggest obtaining at least 4 biopsy fragments containing cancer in endoscopic biopsy for accurate HER2 test and recommend to record tumor fragments number in HER2 IHC pathologic report.
